# Minimally invasive approach to cervical intraspinal air gun pellet in a pediatric patient

**DOI:** 10.1007/s00381-025-07068-w

**Published:** 2025-12-05

**Authors:** Olivia F. Patch, John H. Wilkinson, Joaquin A. Hidalgo

**Affiliations:** Department of Neurosurgery, University of Mississippi Medical Center, Jackson, MS USA

**Keywords:** Ballistic injury, Radiculopathy, Pediatrics, Pellet gun injury

## Abstract

**Background:**

The authors report a case of a 12-year-old girl who presented for neurosurgical evaluation one month after sustaining a ballistic injury to the anterior left side of her neck from a pellet gun accidental firing. Immediately following the misfire, the patient experienced pain in the left side of her neck that radiated to her shoulder, down her posterior arm, and to her middle fingers on the left hand. Subsequently, the patient experienced lingering numbness and developed progressive weakness in her left arm. The patient denied trouble swallowing or any other deficits. Radiographic studies of the cervical spine revealed a ballistic fragment lodged in the C6–7 left foramen, and surgical removal was offered considering debilitating neurological deficits and pain. Preoperative imaging including cervical x-rays and CT of the neck were obtained. A posterior left-sided C6–C7 minimally invasive tubular hemilamino-foraminotomy was performed. The projectile was successfully retrieved, and the C7 nerve root was decompressed and preserved.

**Observations:**

Postoperatively, the patient regained normal strength and function of the left hand and continues to report satisfaction with the surgical outcome.

**Lessons:**

This case presentation highlights the surgical management of a C7 radiculopathy due to a retained foreign body in a pediatric patient from a pellet gun accident. It highlights the importance of careful preoperative planning to determine the safest, most efficient, and minimally invasive surgical approach.

## Introduction

The purpose of this article is to describe a rare case of C7 radiculopathy in a pediatric patient following a pellet gun injury, in which a ballistic fragment became lodged in the cervical spine. By detailing the clinical presentation, diagnostic evaluation, and surgical management, this report aims to contribute to the limited body of literature on pediatric penetrating spinal trauma and highlight considerations for timely recognition and intervention in similar cases.

### Illustrative case

A 12-year-old female presented for neurosurgical evaluation for a retained ballistic fragment at the level of the C6-C7 vertebrae. The patient was accidentally shot in the neck with an air gun during a holiday celebration one month prior to presentation. The patient experienced neck pain that radiated to her shoulder and down her left arm as well as numbness and weakness in her left upper extremity. On physical examination, the patient had weakness in her left triceps. She reported that flexion of the fingers of her left hand intensifies the pain. Her neck was supple and non-tender where the bullet entered her anterior neck to the left of the midline.

**Fig. 1:**
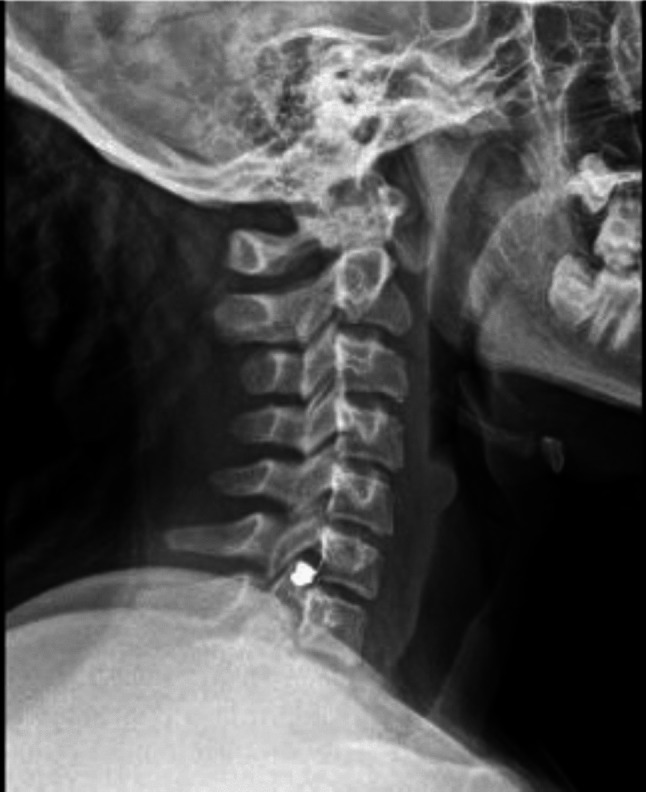
A lateral cervical X-ray reveals the ballistic fragment at the level of the C6–C7 vertebrae

X-rays were obtained, which showed a ballistic fragment to the left of midline and posterior to the disc space of C6–C7 vertebrae, adjacent to the left pedicle of C7 (Fig. 1).

**Fig. 2:**
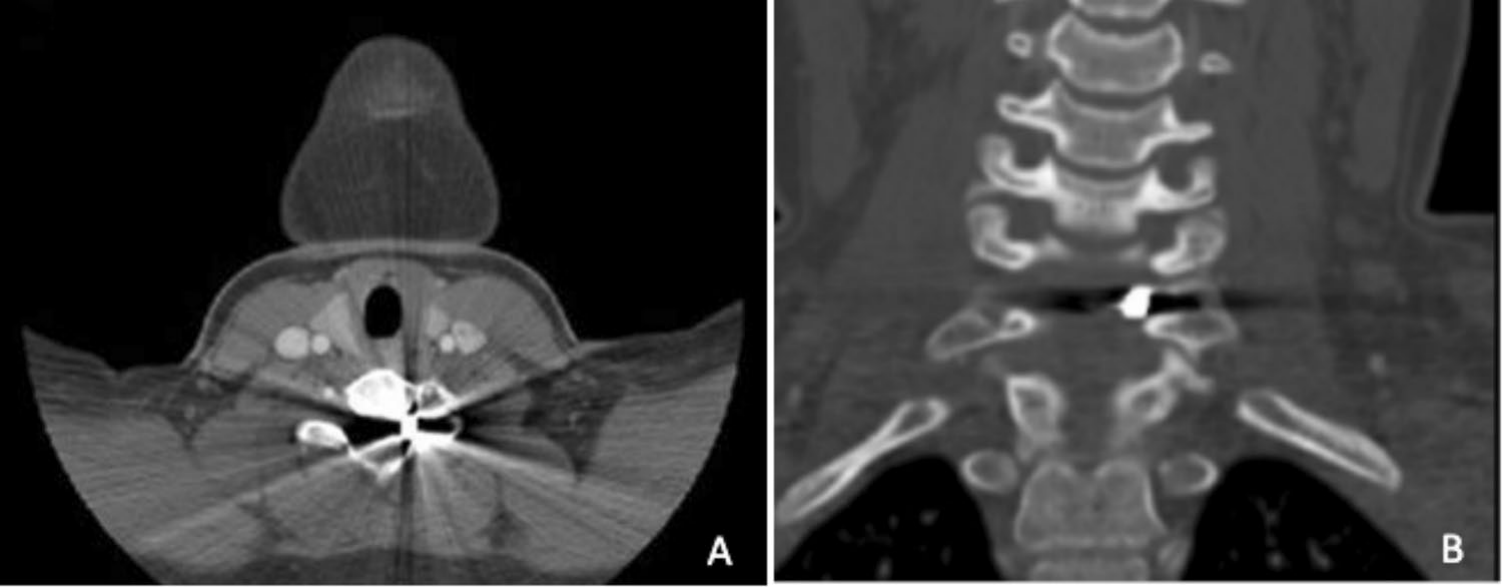
Preoperative CTA confirming the location of the pellet. **A** Axial view. **B** Coronal view

No acute fracture or soft tissue damage was appreciated. A CT/CTA was performed, evidencing the location of the fragment as well as ruling out vascular damage (Fig. 2). The patient and her guardian were presented with two therapeutic options: follow up with serial clinical and radiological imaging or surgical removal of the fragment in light of symptomatic radiculopathy with motor weakness. The patient and her guardian decided to proceed with surgical excision of the foreign body given the debilitating symptoms.

Both an anterior and a posterior approach were considered during surgical planning. The anterior approach presented several disadvantages, including intervertebral disc removal and instrumented fusion in the absence of spinal instability in a pediatric patient. The posterior approach, in turn, could be performed with a hemilaminectomy without the need for fusion, and this was the elected approach.

**Fig. 3:**
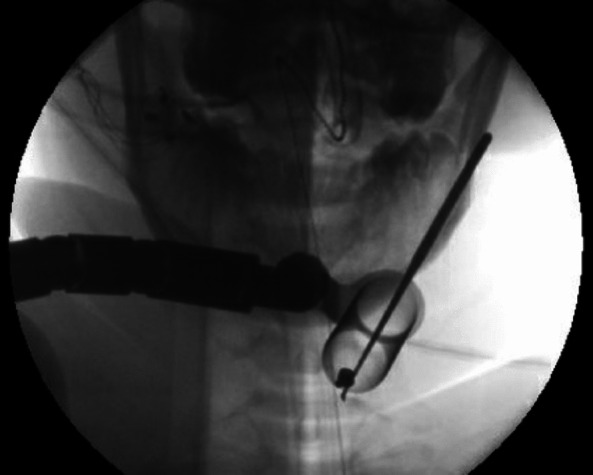
Intraoperative AP X-ray showing pellet lying within working channel

The patient was taken to the OR for surgical removal of the ballistic fragment. The patient was placed under general endotracheal anesthesia and positioned prone on chest rolls and carefully padded extremities. Prepositioning SSEP, MEP, and EMG were obtained, and they were consistent with left upper extremity findings in prior exams. Her head was secured using the 3-point pin fixation. The medial aspect of the C6–C7 facets was localized using AP and lateral fluoroscopy, at which point a 3 cm incision line was drawn. The patient was prepped and draped in the standard sterile fashion, and a sharp incision was made along the previously described line. Under lateral fluoroscopic guidance, progressive tubular dilators were inserted on the inferomedial aspect of the left C6–C7 lamina-facet transition (Fig. 3).

**Fig. 4:**
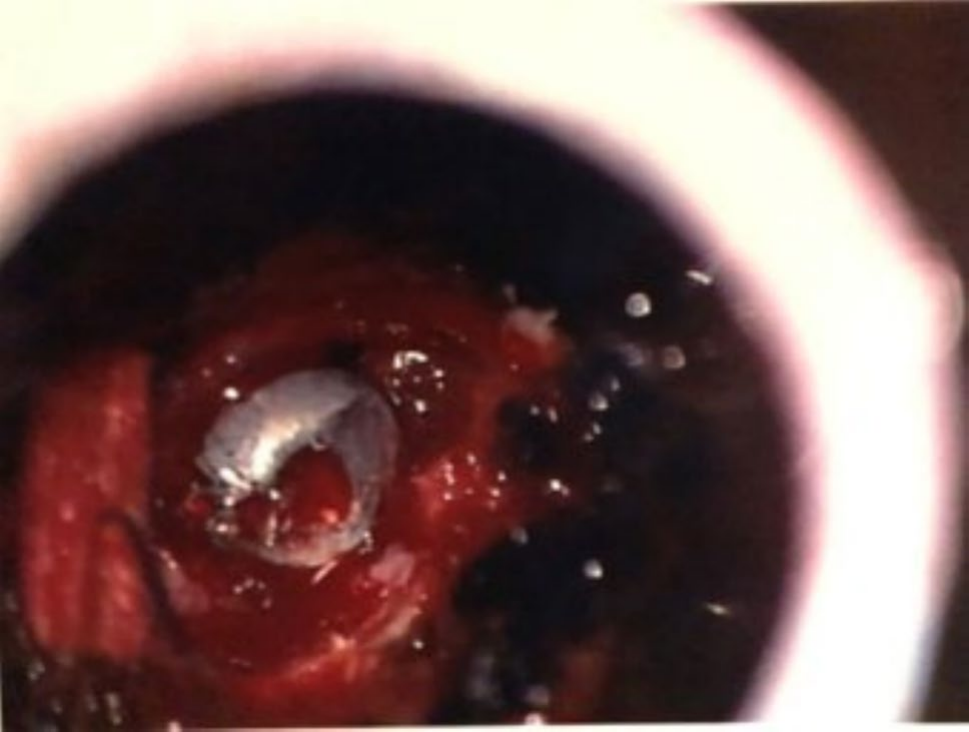
Intraoperative photograph taken through the microscope showing the pellet after dissection from surrounding swollen tissue and before removal from the surface of the C7 nerve root

A tubular working channel was inserted into the dilator and locked into position over the bone. A high-speed drill was then used to perform the C6 and C7 hemilaminotomies, and then the ligamentum flavum was breached using a small nerve hook and 1 and 2 mm Kerrison punches. A left C6–C7 limited medial facetectomy was performed. The projectile was located adjacent to the C7 nerve root in the foramen, causing compression of the root. It was carefully dissected from inflamed tissue and removed, successfully freeing the compressed C7 nerve root (Fig. 4). The nerve root itself appeared to be inflamed yet grossly undamaged.

Culture swabs were obtained, and the wound was abundantly irrigated. Closure was performed in layers using interrupted 3.0 Vicryl for the dermis and nylon for the incision line. The wound was dressed using standard post-operative bandages. The procedure concluded without complication.

## Discussion

Pellet gun injuries are relatively common in the pediatric population, but they rarely lead to devastating neurological outcomes. Such injuries in children that do result in neurological consequences most commonly occur via entry points through the orbit or soft tissues in the neck with subsequent vascular damage [1]. In this case, we describe a unique presentation of cervical radiculopathy resulting from a penetrating pellet gun injury in a child—an injury mechanism more frequently associated with soft tissue trauma in the adult population than with neurologic sequelae in pediatrics [2]. While the adult skeleton can stop these low-velocity projectiles, the thin bones of young children can easily be compromised [2]. Several cases exist detailing pellet gun fragments getting lodged in the spine canals of children, piercing the soft bones of the skull in infants, or transversing the orbit [1, 2]. Although sometimes clinically silent at the time of injury, any potential penetrating injury from a pellet gun in a child should prompt immediate evaluation. Spinal injuries in particular can be overlooked during the initial exam as some wounds tend to appear strictly superficial [2].

For penetrating spinal injuries, some authors argue for early surgery given the high risk for meningitis and the possibility of other complications developing over time, such as adhesions or late spinal-cord injury. In the case of a 2-year-old male who had accidentally been shot in the back of the neck with a pellet gun, surgical management was the favored approach given the high concern for meningitis. Initially, the parents did not seek medical attention as the patient was asymptomatic and they believed the injury to be superficial. However, four days after the injury, the patient developed decreased neck mobility, prompting a medical evaluation. Cervical radiographs and CT imaging showed the pellet within the spinal canal, and surgical removal of the fragment was achieved via an intralaminar approach. It was discovered that the bullet had pierced the ligamentum flavum and dura mater and was lying on the surface of the spinal cord. Postoperative recovery was unremarkable, and the patient remained neurologically intact [2].

In the scenario of asymptomatic spinal penetration injuries, there are cases of successful conservative management yielding favorable outcomes. One such case is that of a 6-year-old boy accidentally shooting himself with an air gun through the mouth, resulting in the bullet lodging in his C1 vertebrae. On examination, the patient was hemodynamically stable and neurologically intact. Treatment options were discussed with the family, but given the asymptomatic nature of the patient’s injury as well as financial concerns, conservative management with close outpatient follow-up was considered the best option. Serial radiographic imaging revealed no change in the pellet’s location. Ultimately, the patient continued to do well, and at one year follow-up, remained asymptomatic with no neurological or developmental issues [3].

Cervical radiculopathies can occur via direct compression or contusion of the nerve root, local hematomas, or inflammatory reactions. Clinically, C7 radiculopathies can be difficult to differentiate from C6 radiculopathy, given the overlap in symptoms [4]. However, Rainville et al. reported that on physical examination, patients with C7 radiculopathy were more likely to report heightened pain sensation and greater symptom frequency in the posterior forearm and the posterior index finger than those with C6 radiculopathies. While there are several etiologies of clinical cervical radiculopathy, compression of cervical nerve roots is seen as one of the most common causes [5]. In adults, this is frequently caused by disc herniation, osteophyte formation, or foraminal stenosis as a result of disc space narrowing [5]. In children, cervical radiculopathies can be observed following trauma, but rarely as isolated pathologies as in the case of our patient. For our patient, the bullet followed a trajectory that avoided any vascular damage to the neck and avoided contact with the spinal cord.

In our patient’s case, several factors had to be taken into consideration before proceeding with surgery. One factor was that the patient’s high BMI and somewhat difficult body habitus made localization somewhat challenging. Radiological localization was only possible in AP fluoroscopy views. A salvage plan for localization in our institution would be intraoperative CT of the spine with navigation. However, it was not necessary in this case.

In the setting of existing vascular injury, the posterior approach would have also been advantageous in isolating the vertebral artery for repair or ligation purposes. Preoperative CTA of the neck was obtained particularly to screen for a vascular injury and decide, depending on the type of injury, if an interventional procedure or anticoagulation/antiplatelet therapy was necessary before surgery and to weigh the risks versus benefits of surgical intervention. In the setting of debilitating radiculopathy, even in the presence of a vascular injury, removal could have still been possible depending on the type and severity of the injury.

### Observations

Although the patient experienced a relatively benign clinical course following what could have been a devastating spinal injury, her persistent neurologic symptoms—namely paresthesia and progressive upper extremity weakness—were unlikely to resolve without intervention. Conservative management was considered and undertaken for over 1 month; however, the severity and progression of her deficits, combined with the fragment’s proximity to the left C7 nerve root, raised concern for ongoing neural irritation, compression, or future complications such as fibrosis or permanent nerve dysfunction. These factors ultimately led to the decision to pursue surgical removal of the ballistic fragment via a posterior approach, which offered safe access while avoiding the morbidity associated with anterior or posterior cervical fusion.

Postoperatively, the patient demonstrated an excellent neurologic recovery. She was discharged within 24 h, and at follow-up visits, she showed full (5/5) motor strength in both upper extremities, resolution of paresthesia, and the return of painless finger flexion. With continued outpatient physical therapy, her fine motor coordination and grip strength improved further, allowing her to return to normal daily activities without restrictions.

### Lessons

This case highlights the importance of thorough neurological evaluation and imaging in pediatric patients presenting persistent pain or weakness following penetrating trauma, even from low-velocity projectiles such as pellet guns. While these injuries may initially appear minor, retained ballistic fragments near critical neurovascular structures can result in delayed but clinically significant deficits. Early recognition of radiculopathy symptoms and careful surgical planning—particularly when weighing anterior versus posterior approaches—can lead to excellent functional recovery. For our patient, proceeding with a posterior approach allowed her to recover full mobility of her left upper extremity while preserving normal range of motion of the cervical spine by avoiding instrumental fusion that would have been necessary with an anterior approach.

## Data Availability

No datasets were generated or analysed during the current study.

## Abbreviations

CTA: Computed tomography angiogram
SSEP: Somatosensory evoke potentials
MEP: Motor evoked potentials
EMG: Electromyography
